# Unveiling the Impact of Eco-Friendly Synthesized Nanoparticles on Vegetative Growth and Gene Expression in *Pelargonium graveolens* and *Sinapis alba* L.

**DOI:** 10.3390/molecules29143394

**Published:** 2024-07-19

**Authors:** Maha M. Kamel, Abdelfattah Badr, Dalal Hussien M. Alkhalifah, Rehab Mahmoud, Yasser GadelHak, Wael N. Hozzein

**Affiliations:** 1 Botany and Microbiology Department, Faculty of Science, Beni-Suef University, Bani Suef 62521, Egypt; mahamohamed@science.bsu.edu.eg (M.M.K.); hozzein29@yahoo.com (W.N.H.); 2 Botany and Microbiology Department, Faculty of Science, Helwan University, Helwan, Cairo 11790, Egypt; 3 Department of Biology, College of Science, Princess Nourah bint Abdulrahman University, P.O. Box 84428, Riyadh 11671, Saudi Arabia; 4 Chemistry Department, Faculty of Science, Beni-Suef University, Beni Suef 62511, Egypt; 5 Department of Materials Science and Nanotechnology, Faculty of Postgraduate Studies for Advanced Sciences, Beni-Suef University, Beni Suef 62511, Egypt

**Keywords:** green synthesis, *Allium cepa* root, germination percentage, Mg NP/GW, *Pelargonium*, fertilizer

## Abstract

Nanoscale geranium waste (GW) and magnesium nanoparticle/GW nanocomposites (Mg NP/GW) were prepared using green synthesis. The Mg NP/GW samples were subjected to characterization using X-ray diffraction (XRD) and Fourier-transform infrared spectroscopy (FTIR-FT). The surface morphology of the materials was examined using a scanning electron microscope (SEM), and their thermal stability was assessed through thermal gravimetric analysis (TG). The BET-specific surface area, pore volume, and pore size distribution of the prepared materials were determined using the N2 adsorption–desorption method. Additionally, the particle size and zeta potentials of the materials were also measured. The influence of the prepared nanomaterials on seed germination was intensively investigated. The results revealed an increase in seed germination percent at low concentrations of Mg NP/GWs. Upon treatment with Mg NP/GW nanoparticles, a reduction in the mitotic index (MI) was observed, indicating a decrease in cell division. Additionally, an increase in chromosomal abnormalities was detected. The efficacy of GW and Mg NP/GW nanoparticles as new elicitors was evaluated by studying their impact on the expression levels of the farnesyl diphosphate synthase (FPPS1) and geranylgeranyl pyrophosphate (GPPS1) genes. These genes play a crucial role in the terpenoid biosynthesis pathway in *Sinapis alba* (*S. alba*) and *Pelargonium graveolens* (*P. graveolens*) plants. The expression levels were analyzed using reverse transcription–quantitative polymerase chain reaction (RT-qPCR) analysis. The qRT-PCR analysis of FPPS and GPPS gene expression was performed. The outputs of FPPS1 gene expression demonstrated high levels of mRNA in both *S. alba* and *P. graveolens* with fold changes of 25.24 and 21.68, respectively. In contrast, the minimum expression levels were observed for the GPPS1 gene, with fold changes of 11.28 and 6.48 in *S. alba* and *P. graveolens*, respectively. Thus, this study offers the employment of medicinal plants as an alternative to fertilizer usage resulting in promoting environmental preservation, optimal waste utilization, reducing water consumption, and cost reduction.

## 1. Introduction

In recent times, there has been a growing recognition of the environmental challenges posed by various factors such as climate change, water contamination, finite natural resources, and their implications for human well-being [1]. Consequently, there has been an increased emphasis on developing eco-conscious products and processes. In line with this perspective, researchers have been actively exploring various approaches to enhance the production of metal and metal oxide nanoparticles using more sustainable technologies [2]. Utilizing biological substrates for the synthesis of nanostructured metal and metal oxides is an intriguing approach that has gained significant attention as a potential alternative to conventional methods prevalent across various industries [3].

The use of medicinal plants is seen as a promising link between economic development, accessible healthcare, and the conservation of vital biodiversity [4]. As Larsen and Olsen [5] argue, “Millions of people rely on medicinal plants to meet some or all of their healthcare needs”. Thousands of plant species are utilized, with medicinal applications potentially being the most widespread form of biodiversity utilization. Focusing on the challenge of fostering sustainable economic growth in remote areas, medicinal plants offer marginalized rural communities a viable avenue for income generation [6]. Leaves are extensively used in traditional medicine as well as for culinary and tea enhancements.

The development of environmentally friendly processes for nanoparticle synthesis is emerging as a prominent field within nanotechnology [7,8]. Green nanotechnology aims to enable the production of commercially viable, environmentally friendly, and safer products based on nanotechnology. This area is currently receiving increased attention, particularly in the “green synthesis” of metal nanoparticles, which exhibit unique optical, chemical, photochemical, and electrical properties [9]. Eco-friendly biosynthesis of nanoparticles is gaining traction in response to the demand for environmentally conscious material synthesis [10,11,12]. Over the past decade, there has been a notable surge in interest in nanoparticles due to their increasing use in electronics, micronutrients for plants and biofertilizers, and environmental remediation [13]. The growing use of nanoparticles highlights the importance of understanding their environmental impact [14].

Compared to microorganisms, plants offer advantages in terms of the speed and stability of nanoparticle synthesis. Additionally, plant-based synthesis yields nanoparticles with a wider range of sizes and shapes compared to those produced by other organisms. Researchers are investigating the mechanisms of metal ion uptake, bioreduction by plants, and potential pathways for metal nanoparticle formation within plants, driven by the benefits of using plants and plant-derived materials for the biosynthesis of metal nanoparticles [15,16,17,18]. The reproducibility of nanoparticles synthesis using plant extracts is influenced by various factors such as the presence of phytochemicals in the extracts acting as stabilizing and reducing agents, the type of plant species used, and the optimization of reaction conditions [19,20,21]. Plant extracts contain biomolecules like alkaloids, flavonoids, saponins, and tannins that aid in reducing metal ions and creating nanoparticles, leading to a single-step synthesis process [20,22]. Studies have shown that the synthesis of silver nanoparticles using *mangosteen pericarp* ethanolic extract resulted in reproducible nanoparticles with sizes ranging from 7 to 38 nm, showcasing the potential for consistent synthesis outcomes [22]. The green synthesis approach using plant extracts offers a sustainable and environmentally friendly method for nanoparticle production, highlighting the reproducibility and reliability of this technique in creating nanoparticles for various applications [20,21].

In recent decades, inorganic materials such as metal and metal oxides have garnered significant attention. One promising and environmentally friendly approach to producing inorganic metal nanoparticles is through the green synthesis of Mg NP/GW nanocomposites. These nanoparticles exhibit superior qualities, including a higher stability-to-weight ratio, fewer defects, recyclability, non-toxicity, and hygroscopicity. These properties enable them to withstand harsh processing conditions and offer intriguing structural properties for biological applications [23,24]. However, despite these commendable attributes, Mg NP/GW nanoparticles often have large crystalline sizes and low surface areas, which limit their practical applications. Addressing this limitation requires morphological investigations aimed at modifying surface area in relation to particle size [25,26,27].

Rose-scented geranium (*Pelargonium graveolens*), a perennial aromatic and medicinal herb from the Geraniaceae family, is a significant plant known for producing essential oil (EO) rich in monoterpenes and sesquiterpenes among other compounds. The Geraniaceae family comprises approximately 750 species found in temperate and subtropical regions [28]. The EO derived from *P. graveolens* finds economic applications in perfumery, flavoring, and cosmetics [29,30]. The production of nanoparticles (NPs) using residual material from *Pelargonium graveolens* (geranium) and subsequent analysis on the same plant is driven by various scientific incentives, including sustainability and resource utilization. This approach aims to promote sustainable methodologies in nanotechnology by transforming agricultural waste into valuable NPs, reducing environmental impact, and promoting circular bioeconomy principles. Additionally, the compatibility and potential toxicity of these NPs on *P. graveolens* are crucial for safe and effective agricultural applications, ensuring that the NPs do not harm plant vitality or development. This aligns with the principles of green chemistry and sustainable nanotechnology, which aim to minimize the use of harmful substances and reduce the ecological impact of NP production. By examining the effects on the same plant species, the environmental soundness of this method can be confirmed.

Geranium waste (GW) serves as a natural binding agent in nanoparticle synthesis and holds promise for enhancing crop production, making it a foundational element in science-based agriculture essential for sustaining the growing global population. The term “waste (GW) originating from the *Pelargonium graveolens* plant” refers to the residual materials or byproducts that are produced during the cultivation, harvesting, and processing of *P. graveolens*, also known as geranium. The main purpose of growing this plant is to extract its essential oil, which is used in various industries such as perfumery, cosmetics, and aromatherapy. The waste material, known as geranium waste (GW), includes the following: Residues of leaves and stems—After the essential oil is extracted, the remaining plant biomass, which consists of leaves and stems, is considered waste. This biomass is usually discarded or used for composting. Non-essential oil-containing components—Parts of the plant that are not involved in the oil extraction process or do not contain significant amounts of essential oils are classified as waste. Trimming and cutting debris—During cultivation and harvesting, excess or unwanted parts of the plant, such as trimmings and cuttings, are produced and considered waste. The term “waste” is used because these materials are typically not used in the primary production process (extraction of essential oil) and are often discarded or repurposed for low-value purposes like composting or animal feed. However, this “waste” still contains valuable bioactive compounds and biomass that could be repurposed for alternative applications, such as the production of nanoparticles.

Another plant, *Sinapis alba* (*S. alba*), also known as white or yellow mustard, originates from the Mediterranean and is an annual plant. White mustard seeds are valued in agriculture for their high protein and oil content and low carbohydrate content [31]. They serve as a valuable source of high-quality proteins due to their balanced composition of amino acids. In various applications such as meat processing, salad dressings, mayonnaise, and as extenders in protein, they are frequently employed as binding agents. Additionally, their potent antibacterial and disinfecting properties make the seeds useful in food preservation [32,33].

## 2. Results and Discussion

### 2.1. Characterization of Nanomaterial

**Figure 1:**
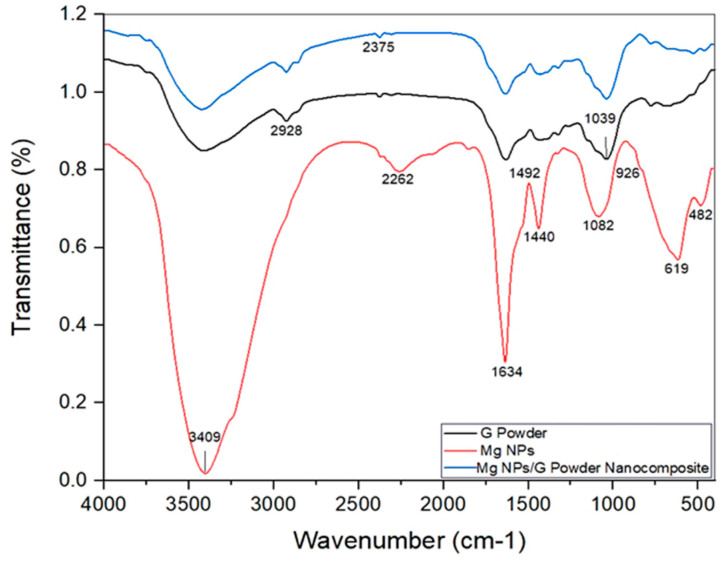
FTIR spectra of GW, Mg NPs, and Mg NP/GW powder nanocomposite.

The FTIR spectra of the GW, Mg NPs, and Mg NP/GW nanocomposite are depicted in Figure 1. Notable bands were observed at 3409 cm^−1^, 2928 cm^−1^, 2375 cm^−1^, 1634 cm^−1^, and 1039 cm^−1^ for the nanocomposite. In the Mg NP sample, the peak at 3409 cm^−1^ may be attributed to the O-H stretching vibrations of adsorbed water molecules. The band at 2262 cm^−1^ can be assigned to a nitrile group (C≡N), possibly originating from nitrate reduction during the synthesis processes [34,35]. The sharp peak at 1634 cm^−1^ can be attributed to the aromatic C=C stretching vibrations [36]. Additionally, the peaks at 1440 cm^−1^ and 1062 cm^−1^ may correspond to amide II and C-O single bond, respectively [37]. 

The peaks observed for the Mg NP/GW nanocomposite are a combination of the specific peaks seen in the GW and Mg NP samples. Furthermore, the low-intensity peaks at 926, 619, and 482 cm^−1^ can be attributed to the metal oxide bond formation (M-O, M-O-M, O-M-O) at the surface of the magnesium nanoparticles.

**Figure 2:**
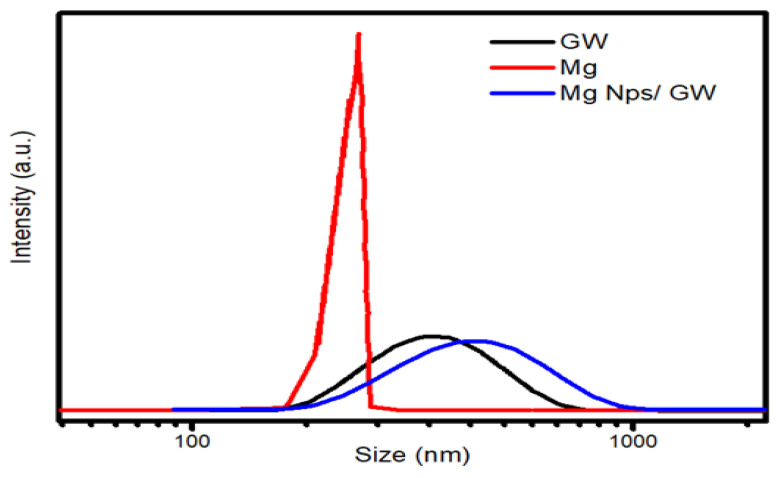
Hydrodynamic size of GW, Mg NPs, and Mg NP/GW powder nanocomposite.

The hydrodynamic sizes of the produced samples are illustrated in Figure 2. The GW sample exhibited a broad peak with maximum intensity at approximately 400 nm. Following Mg precipitation in the Mg NP/GW nanocomposite, a slight shift towards larger sizes was observed, indicating an increase in hydrodynamic size after Mg precipitation. Conversely, the zero-valent Mg sample displayed a sharp peak at approximately 250 nm.

**Figure 3:**
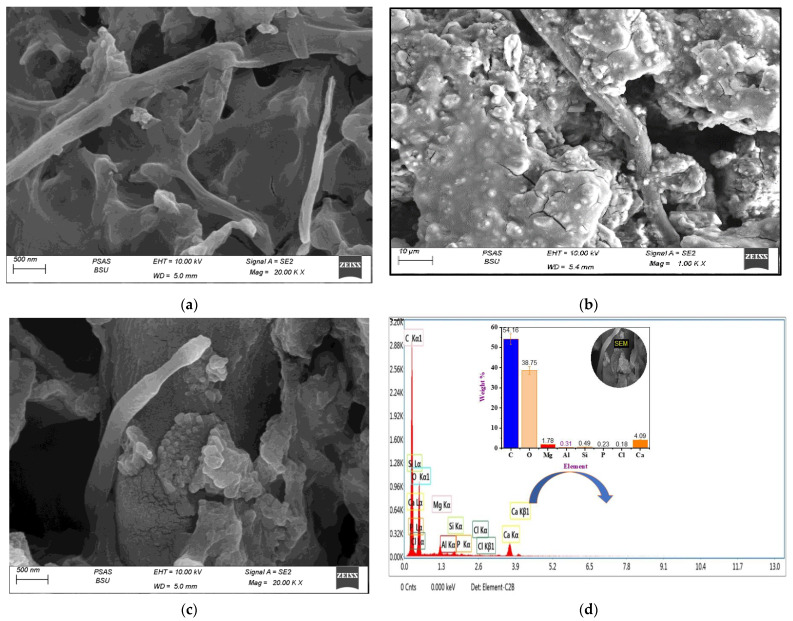
SEM images of (**a**) GW, (**b**) Mg NPs, (**c**) Mg NP/GW powder nanocomposite; (**d**) EDX of Mg NP/GW powder nanocomposite.

Figure 3a depicts an SEM image of the GW sample, illustrating long fibrous materials typical of powdered plant-based samples. In Figure 3b, Mg nanoparticles are visible, exhibiting extensive aggregation and entanglement within the extracted matrix. Such aggregation is common in zero-valent metals synthesized through green methods, particularly when the metal concentration is significantly lower than that of the extract, as observed in this image.

Figure 3c shows the composite where Mg nanoparticles are aggregated over the GW powder composed of fibrous material (Figure 3a). Figure 3d presents the EDX results for the Mg NP/GW powder nanocomposite. The composite primarily comprises carbon and oxygen originating from the GW powder. Additionally, other elements such as calcium, aluminum, and silicon are detected. The atomic percentage of magnesium in the composite is approximately 1% of the total elements present, while carbon and oxygen combined represent more than 96% of the total elements present.

**Figure 4:**
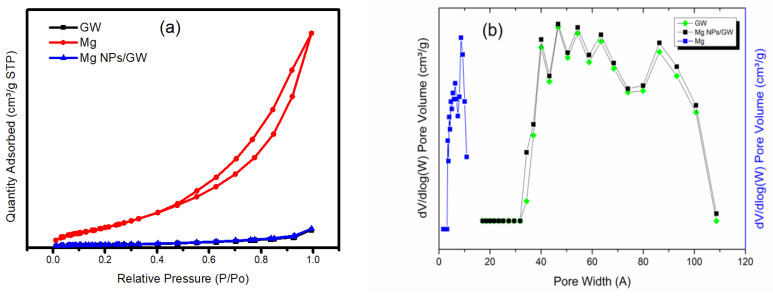
(**a**) N_2_ adsorption–desorption isotherms and (**b**) pore size distribution of GW, Mg NPs, and Mg NP/GW powder nanocomposite.

Figure 4a illustrates nitrogen adsorption–desorption isotherms classified as type IV. Among the samples, the Mg sample exhibited the highest surface area, measured at 42.2 m^2^/g as tabulated in Table 1.

Figure 4b displays the pore size distribution estimated from the nitrogen desorption branch of the isotherm. Notably, the Mg sample demonstrated the narrowest pore size distribution, with an average pore diameter of 0.5 nm (Table 1). In contrast, both the GW and composite samples exhibited similar wide pore size distributions, as depicted in Figure 4b.

**Figure 5:**
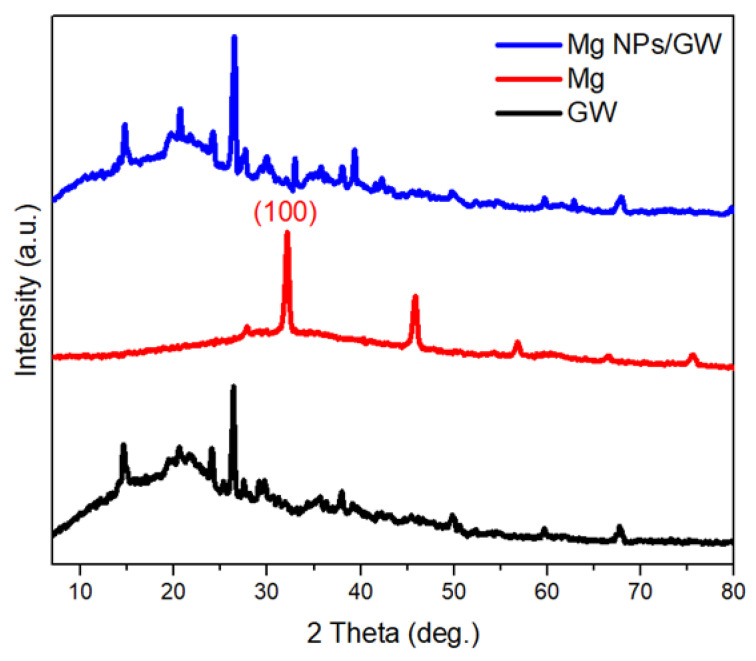
XRD of GW, Mg NPs, and Mg NP/GW powder nanocomposite.

Figure 5 illustrates the XRD patterns of GW, Mg NPs, and Mg NP/GW powder nanocomposite. In the Mg sample, the main peak observed at 32° corresponds to the (100) plane family of Mg [38]. Importantly, no peaks corresponding to magnesium oxide (MgO) were detected, suggesting that any observed metal oxide bonds, such as Mg-O, Mg-O-Mg, and O-Mg-O, likely form on the surface of the Mg nanoparticles rather than in their core. This interpretation is supported by the absence of MgO peaks in the XRD diffractogram. The peaks observed for the GW sample can be attributed to the presence of numerous organic compounds within the GW powder matrix. In the composite sample, peaks from both the GW and Mg NPs are evident, indicating the presence of Mg nanoparticles supported on the GW powder. However, the peaks from GW are dominant, likely due to the lower weight percentage of Mg in this composite. Based on XRD data and using the Scherrer equation, the crystallite sizes were estimated to be 21.6 nm for Mg NPs and 21.3 nm for the Mg NP/GW nanocomposite.

**Figure 6:**
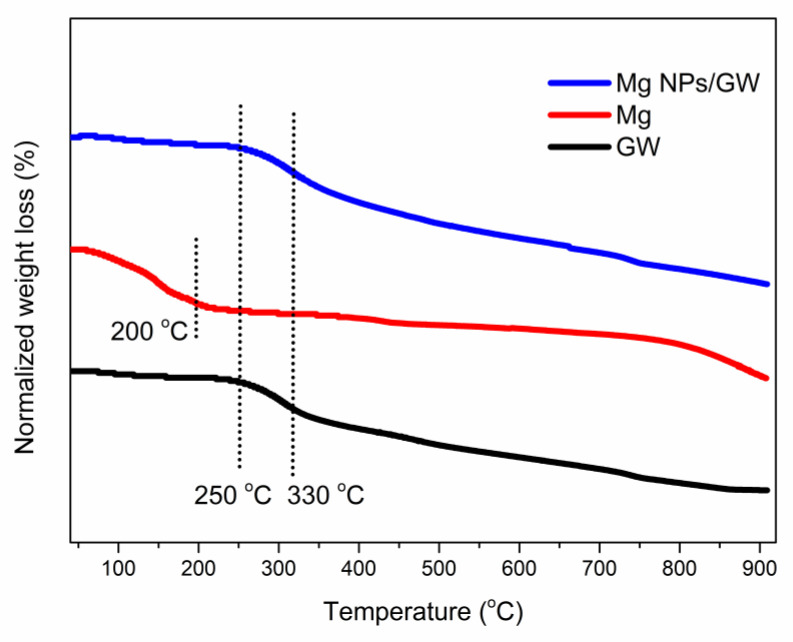
TGA of GW, Mg NPs, and Mg NP/GW powder nanocomposite.

Figure 6 presents the TGA analysis of the prepared samples. A gradual weight loss is observed for the GW sample up to 250 °C, attributed to the evaporation of water vapor and volatile organic compounds from the GW matrix. Between 250 °C and 330 °C, a noticeable weight loss occurs, corresponding to the decomposition of organic molecules within the GW powder [39]. This decomposition continues up to 900 °C, depending on the temperature required to evaporate the larger organic molecules.

A similar trend is observed for the Mg NP/GW powder nanocomposite samples. However, the Mg sample displays a different trend, with an observable weight loss occurring up to 200 °C. This weight loss is likely due to the evaporation of water molecules and volatile compounds attached to the surface of the Mg nanoparticles. The transition from Mg to MgO is not observable, possibly due to the low concentration of Mg compared to the extract.

### 2.2. Effect of Nanoparticles Mg NP/GW and GW NPs on Sinapis alba Seed Germination

The impact of Mg NP/GW and GW NP applications on *S. alba* seed germination indicates that at lower concentrations of Mg NP/GW, seed germination and seedling growth increased, while at higher concentrations, these processes were inhibited (Table 2). Specifically, seeds treated with GW (0.5 g/L) exhibited a seed germination rate of 96.67%, whereas the lowest germination percentage was observed in the GW (5.0 g/L) treatment, with only 46.67% germination. Similarly, the highest concentration (5.0 g/L) of Mg NP/GW resulted in significantly lower germination percentages, with only 46.67% germination. The maximum seed germination rate was observed at the (0.5 g/L) concentration of Mg NP/GW, reaching 100%.

Overall, the germination percentage declined as NP concentrations increased, indicating an inhibitory effect with higher NP concentrations. These findings suggest that increasing NP concentration leads to decreased seed germination (Table 2). Notably, the control treatment exhibited a 100% germination rate [40]. The lowest concentration of Mg NP/GW (0.5 g/L) demonstrated a significant increase in germination percentages and seedling growth, indicating a potential positive effect. However, the mechanism underlying this positive effect requires further investigation [41,42]. Conversely, the highest concentration of Mg NP/GW (5.0 g/L) resulted in a significant decrease in germination percentages, suggesting a potentially toxic effect on the seeds. This toxicity could stem from various factors, including altered physiological processes or interference with cellular structure [43,44].

Furthermore, the results suggest that the growth-promoting agent GW, at a concentration of 0.5 g/L, can significantly enhance seed germination. This implies that GW may possess properties conducive to seedling development, particularly when combined with the optimal concentration of Mg NP/GW [45]. Further exploration is warranted to elucidate the underlying mechanisms and potential applications of these effects.

### 2.3. Morphological Parameters of Pelargonium graveolens

**Figure 7:**
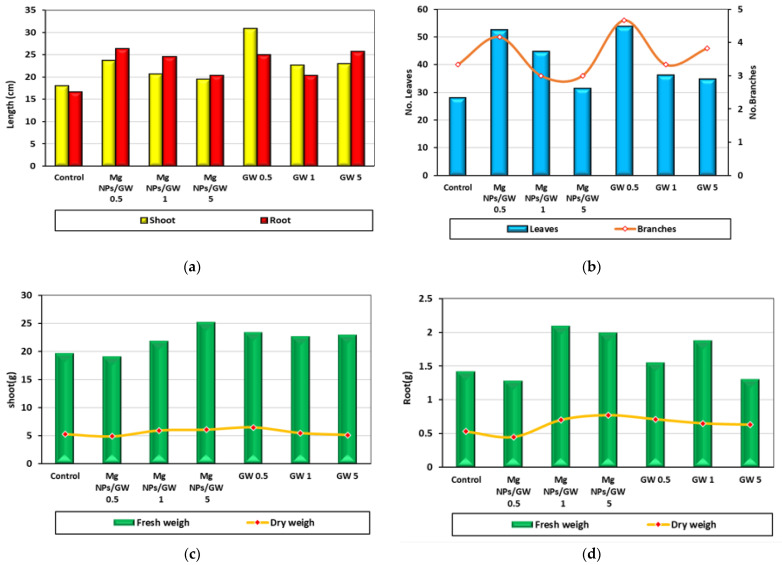
Effect of Mg NP/GW at concentrations of 0.5, 1, and 5, and GW at concentrations of 0.5, 1, and 5 on plant growth and biomass: (**a**) shoot length and root length; (**b**) number of leaves and branches; (**c**) shoot fresh weight and dry weight; (**d**) root fresh weight and dry weight of *P. graveolens*.

The effects of GW and Mg NP/GW on the morphological growth of *P. graveolens* were assessed by measuring plant fresh mass, dry mass, root–shoot length, leaf fresh mass, leaf dry mass, number of leaves, and branch counts (Table 3; Figure 7). Significant differences were observed in these traits in response to treatments with GW and Mg NP/GW at various concentrations.

In terms of shoot length, GW was found to be more effective than Mg NP/GW. However, both the (0.5 g/L) Mg NP/GW and (0.5 g/L) GW treatments resulted in higher numbers of leaves and branches. Additionally, the (0.5 g/L) concentration for both Mg NP/GW and GW resulted in the highest fresh and dry shoot weights. These findings highlight the differential effects of GW and Mg NP/GW on various morphological parameters of *P. graveolens*, suggesting potential applications for optimizing plant growth and development.

**Figure 8:**
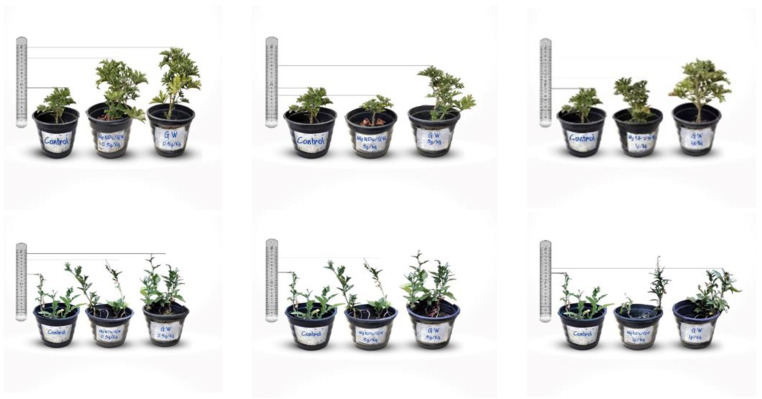
Photographs of plants illustrating the effect of three concentrations (0.5, 1.0, and 5.0 g/L) of Mg NP/GW and GW on the growth of *P. graveolens* (**above**) and *S. alba* (**below**).

Figure 8 illustrates the effect of three concentrations (0.5, 1.0, and 5.0 g/L) of Mg NP/GW and GW on the growth of *P. graveolens* (above) and *S. alba* (below). At low concentrations (0.5 g/L), both Mg NP/GW and GW positively affect plant growth, with Mg NP/GW showing slightly better performance. At medium concentrations (1.0 g/L), the growth effects plateau, showing no significant improvement over the 0.5 g/L concentration. At high concentrations (5.0 g/L), both treatments result in poor growth, significant stunting, and low leaf density. The data suggest that lower concentrations of Mg NP/GW and GW promote better growth in both *P. graveolens* and *S. alba*, while higher concentrations have a detrimental effect.

### 2.4. Effect of Nanoparticle on Mitotic Division

**Figure 9:**
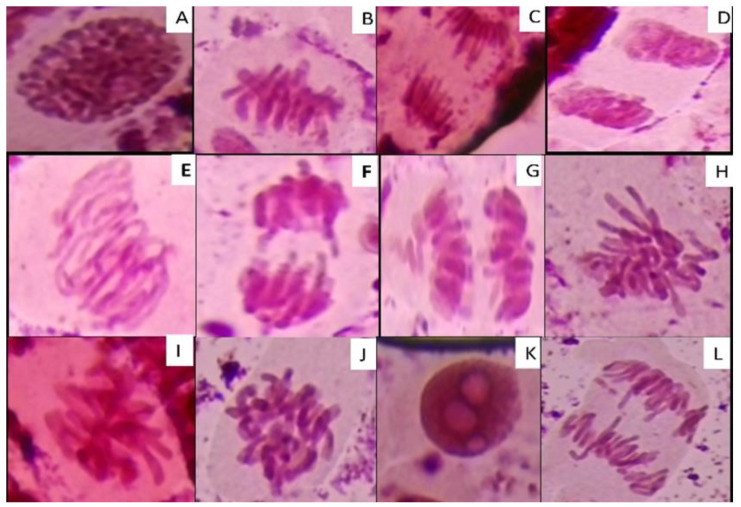
Representative examples of normal and abnormal cell divisions in onion root tips subjected to the various treatments of GW and Mg NP/GW nanoparticles (**A**–**D**): normal prophase, metaphase, anaphase, and telophase, respectively, under control conditions. (**E**): Multiple bridges; (**F**): stickiness in anaphase; (**G**): lagging in anaphase; (**H**): C-shaped metaphase; (**I**): stickiness in metaphase; (**J**): stickiness in metaphase; (**K**): DNA decay; (**L**): breaks and disturbance.

Figure 9 presents normal and abnormal cell divisions in onion root tips exposed to different treatments of GW and Mg NP/GW nanoparticles. In the initial panels (A–D), we observe the typical stages of mitosis in onion root tip cells under control conditions. Panel A shows prophase, where chromosomes condense and become visible, and the nuclear envelope begins to disintegrate. Panel B depicts metaphase, characterized by chromosomes aligning at the cell’s equatorial plate. Panel C illustrates anaphase, where sister chromatids are pulled towards opposite poles. Finally, Panel D shows telophase, where chromatids reach the poles and nuclear envelopes re-form around each set of chromosomes. These stages are essential for ensuring genetic stability during cell division.

The remaining panels (E–L) display various abnormalities induced by treatments with GW and Mg NP/GW nanoparticles. Panel E shows multiple chromosomal bridges, indicating failures in proper chromosome segregation, which can lead to genomic instability. Panel F presents stickiness in anaphase, where chromosomes fail to separate correctly. Panel G shows lagging chromosomes in anaphase, suggesting improper attachment to spindle fibers. Panel H reveals a C-shaped metaphase, indicative of disturbances in chromosome alignment. Panels I and J show stickiness in metaphase, with chromosomes clumping together and failing to align properly. Panel K depicts DNA decay, indicating severe damage. Finally, Panel L displays breaks and disturbances in chromosomes, pointing to significant genotoxic effects.

Table 4 details the effects of different concentrations of Mg NP/GW on mitotic abnormalities. At 0.5 g/L, all abnormalities, including disturbed metaphase, chromosomal bridges, laggards, and sticky chromosomes, are present across the replicates. At higher concentrations (1.0 g/L and 5.0 g/L), the presence of these abnormalities persists, indicating a dose-dependent response. This table underscores the severity and consistency of the abnormalities induced by nanoparticle treatments (Table S1).

Table 4 provides the mitotic index and the distribution of cells across different mitotic stages. As shown in Table S2, under control conditions, the mitotic index averages 44.6%, with a balanced distribution across prophase, metaphase, anaphase, and telophase. However, with nanoparticle treatments, there is a marked increase in the mitotic index and a shift in the distribution. For example, at 0.5 g/L concentration, the mitotic index increases to 61.24%, with significant disturbances in metaphase and anaphase. Higher concentrations (1.0 g/L and 5.0 g/L) show a decrease in the mitotic index, indicating cytotoxic effects that inhibit cell division. These changes reflect the nanoparticles’ disruptive impact on the cell cycle.

Previous studies have established that nanoparticles can exert significant cytotoxic effects on plant cells. For instance, research on silver nanoparticles (AgNPs) demonstrated their ability to disrupt cellular processes and induce oxidative stress, leading to cell cycle arrest and apoptosis [44,46]. Similar findings are reported for titanium dioxide (TiO_2_) nanoparticles, where exposure led to decreased mitotic indices and increased frequencies of chromosomal aberrations in *Allium cepa* root tips [47]. The analysis of the presented figure aligns with these findings, showing a marked increase in mitotic abnormalities and changes in the mitotic index upon treatment with GW and Mg NP/GW nanoparticles. The data in Table 4 supports this by illustrating the variations in the mitotic index and stages of cell division with increasing nanoparticle concentrations.

Nanoparticles are known to cause genotoxic effects, leading to various chromosomal aberrations. Studies have shown that exposure to zinc oxide (ZnO) nanoparticles induces chromosomal stickiness, bridges, and laggards in plant cells, highlighting their potential to disrupt mitotic spindle formation and chromosome segregation [48,49]. The abnormalities observed in the provided figure, such as multiple bridges, stickiness, and lagging chromosomes, are consistent with these genotoxic effects. The quantitative data from Table 4 further corroborate these findings, showing increased frequencies of such abnormalities with nanoparticle treatments.

One of the primary mechanisms through which nanoparticles exert their toxic effects is the generation of reactive oxygen species (ROS). ROS can cause oxidative stress, leading to lipid peroxidation, protein oxidation, and DNA damage [50,51]. This is evident in the study by [52], which demonstrated increased ROS production and DNA strand breaks in plants exposed to various metal nanoparticles. The DNA decay observed in Panel K of the figure can be attributed to such oxidative stress-induced damage. This is supported by the data showing DNA decay percentages in Table 4 which increase with nanoparticle exposure.

Nanoparticles can also interfere with the formation and function of the mitotic spindle, crucial for proper chromosome segregation during cell division. Studies on cerium oxide (CeO_2_) nanoparticles have shown their ability to disrupt microtubule dynamics, leading to spindle abnormalities and mitotic arrest [53]. The presence of C-shaped metaphase and disturbed metaphase in Panels H and L of the figure suggests similar interference with spindle formation. The quantitative representation in Table 4 highlights the prevalence of such disturbances across different nanoparticle concentrations.

### 2.5. Effect of Nanoparticle on Gene Expression

The level of gene expression for the FPPS1 and GPPS1 genes, crucial in regulating the triterpenoid biosynthesis pathway in *P. graveolens* and *S. alba*, was measured using mRNA quantification via qRT-PCR in plants grown under 0.5 g/L of GW NPs and Mg NP/GW.

#### 2.5.1. FPPS1 Gene Expression Analysis Result

**Figure 10:**
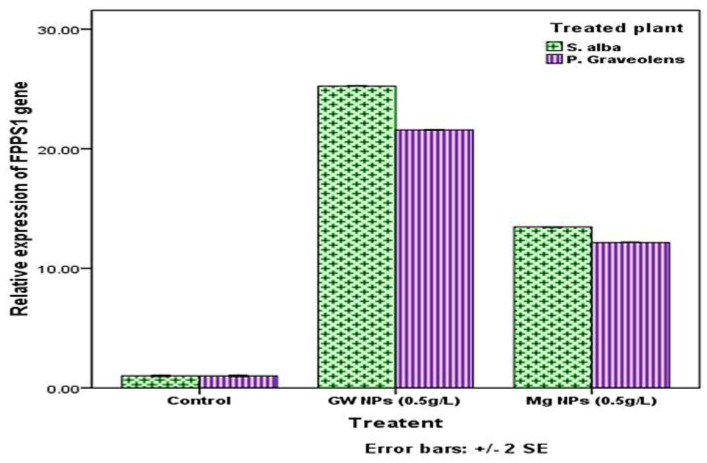
Comparison between (fold changes) of FPPS1 gene in *P. graveolens* and *S. alba* plants growing under (0.5 g/L) GW NP and Mg NP/GW application.

The relative expression levels of the FPPS1 gene were analyzed in the leaves of *P. graveolens* and *S. alba* plants treated with 0.5 g/L of GW NPs and Mg NP/GW (Figure 10 and Table 5). The data revealed an overexpression of the FPPS1 gene in both *P. graveolens* and *S. alba* plants. The highest expression level (25.244 ± 0.004) was observed in *S. alba* plants treated with GW NPs (0.5 g/L), followed by 21.58 ± 0.03 recorded in *P. graveolens* plants treated with GW NPs (0.5 g/L). Additionally, a level of 13.4539 ± 0.002 was observed in *S. alba* plants treated with Mg NP/GW (0.5 g/L) compared to the control group.

#### 2.5.2. GPPS1 Gene Expression Analysis Result

**Figure 11:**
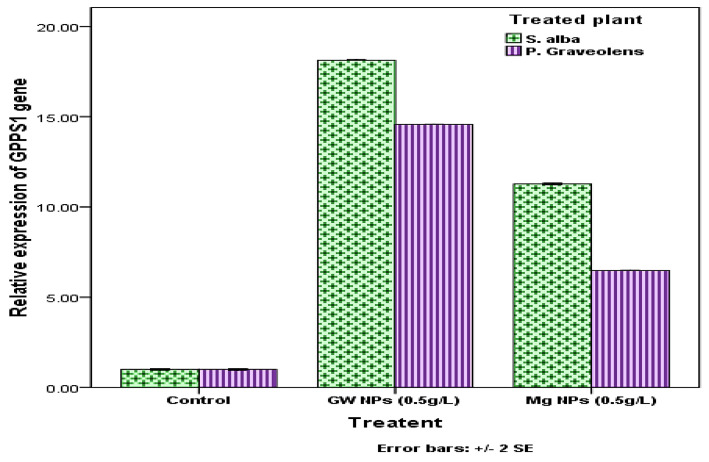
Comparison between (fold changes) of GPPS1 gene in *P. graveolens* and *S. alba* plants growing under (0.5 g/L) GW NP and Mg NP/GW application.

The relative expression levels of the FPPS1 gene were analyzed in leaves of *P. graveolens* and *S. alba* plants treated with GW NPs (0.5 g/L) and Mg NP/GW (Figure 11 and Table 6). Under GW NP (0.5 g/L) treatment, both *S. alba* and *P. graveolens* exhibited significantly elevated fold changes in FPPS1 gene expression compared to the control (*S. alba*: a > b, and *P. graveolens*: b > c). Notably, *S. alba* showed a higher fold change (25.244 ± 0.004) than *P. graveolens* (21.58 ± 0.03) under this condition.

Similarly, under Mg NP (0.5 g/L) treatment, both species showed significantly increased FPPS1 gene expression compared to the control (*S. alba*: c > control, and *P. graveolens*: c > control). However, the fold changes induced by Mg NPs were generally lower than those induced by GW NPs. Once again, *S. alba* exhibited a higher fold change (13.4539 ± 0.002) compared to *P. graveolens* (12.15676 ± 0.0025) under this treatment.

In general, both *S. alba* and *P. graveolens* demonstrated enhanced FPPS1 gene expression in response to GW NP and Mg NP treatments compared to the control. GW NPs induced higher fold changes in gene expression compared to Mg NPs, and *S. alba* consistently exhibited higher fold changes than *P. graveolens* across both nanoparticle treatments.

**Figure 12:**
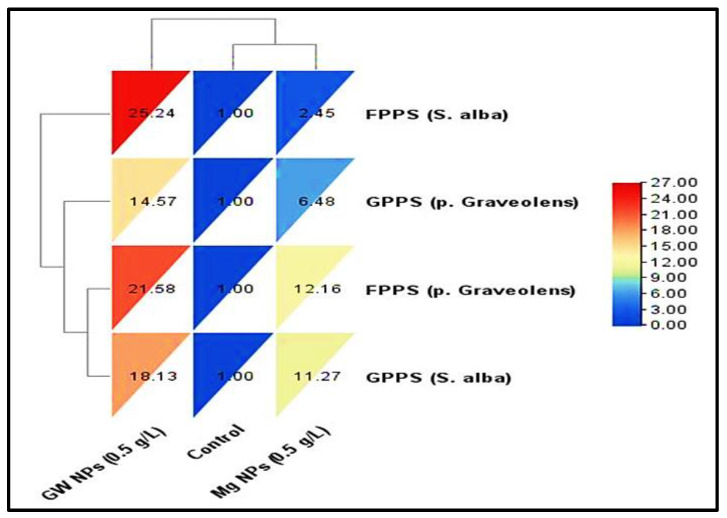
Heatmaps and hierarchical clustering illustrate the results obtained from data of two significantly differential expressed GPPS1 and FPPS1 genes in *P. graveolens* and *S. alba* plants growing under (0.5 g/L) GW NP and Mg NP/GW application.

The heat map in Figure 12 illustrates the results, where each treatment is sorted into two primary clusters: GW- and Mg NP/GW-treated plants in one cluster, and untreated control plants in the other. The highest mRNA expression levels were observed for the FPPS1 gene, with 25.24- and 21.68-fold increases in expression in *S. alba* and *P. graveolens* *, respectively. This was followed by 18.13- and 14.57-fold increases for the GPPS1 gene in *S. alba* and *P. graveolens*, respectively, while 13.45- and 12.16-fold increases were observed for the GPPS1 gene in the same plants. The minimum expression levels were 11.28- and 6.48-fold increases for the GPPS1 gene in the two plants, respectively.

The analysis of RT-qPCR results for FPPS1 and GPPS1 genes in *S. alba* and *P. graveolens* plants revealed interesting findings regarding the mRNA expression levels of these genes in the two plant species. Similar results were reported by [54], showing the effect of silver nanoparticles on the expression of the FAE1 and FAD2 genes in *Camelina sativa* [55].

The mRNA levels of the FPPS1 gene were notably elevated in both *S. alba* and *P. graveolens* plants, with fold changes of 25.24 and 21.68, respectively. These findings highlight a significant upregulation of the FPPS1 gene, underscoring its pivotal role in the biological processes and metabolic pathways of these plant species. This observation is consistent with previous research emphasizing the central role of FPPS1 in plant metabolism.

The study by [56] underscored the importance of specific genes in secondary metabolite biosynthesis pathways, such as thymoquinone in Nigella sativa, demonstrating the relevance of gene expression profiling through RT-qPCR despite challenges posed by residual nanoparticles [57].

In addition to FPPS1, the RT-qPCR results revealed notable fold changes of 18.13 and 14.57 for the expression of the GPPS1 gene in *S. alba* and *P. graveolens*, respectively. While these levels were lower compared to FPPS1, they remain significant, highlighting the importance of GPPS1 in the biochemical pathways of both plant species. Variations in the expression patterns of GPPS1 between *S. alba* and *P. graveolens* may suggest species-specific regulatory mechanisms or environmental influences on gene expression [58].

Conversely, the study documented relatively high expression levels of the FPPS1 gene in both *S. alba* and *P. graveolens*, with fold changes of 13.45 and 12.16, respectively. These findings suggest a critical role for FPPS1 in biological processes, potentially contributing to the production of significant secondary metabolites or engaging in essential physiological functions [59]. In contrast, the GPPS1 gene exhibited lower expression levels, with fold changes of 11.28 and 6.48 in *S. alba* and *P. graveolens*, respectively. These lower levels may indicate a relatively lesser significance of GPPS1 in the examined biological processes or metabolic pathways compared to FPPS1 [60].

Despite the essential role of terpenoids in essential oils, comprising over 80% of their composition, the characterization of genes associated with terpenoid biosynthesis in rose-scented geranium *Pelargonium graveolens* remains limited. Monoterpenes, a class of terpenes with a chemical formula of C10H16, are particularly significant and are synthesized from the precursor geranyl pyrophosphate (GPP) by the enzyme geranyl pyrophosphate synthase (GPPS). These compounds can exhibit acyclic or cyclic structures [61,62].

In plants, terpenoids are synthesized through two primary pathways: the methylerythritol phosphate (MEP) pathway in plastids and the mevalonate (MVA) pathway in the cytoplasm [63,64]. Terpenoid biosynthesis typically progresses through three stages. Initially, two intermediates, isopentenyl diphosphate (IPP) and dimethylallyl diphosphate (DMAPP), are formed, serving as common precursors for terpenoid synthesis and are interchangeable in both cytoplasm and plastids [65]. Subsequently, enzymatic modifications, including methylation, hydroxylation, and glycosylation, contribute to the diverse structures and functions of terpenoids, enriching the array of these compounds found in plants.

### 2.6. Potential Applications of Mg NP/GW Nanoparticles

Magnesium nanoparticles (Mg NPs) and GW nanoparticles hold significant promise for enhancing agricultural productivity. One of the primary applications lies in the development of nanofertilizers, which can increase the bioavailability of essential nutrients to plants. For instance, Mg NPs can boost magnesium uptake, crucial for chlorophyll production and photosynthesis, thereby enhancing plant growth and crop yields. Refs. [66,67] demonstrated that nanoparticles could improve nutrient uptake and utilization efficiency, leading to more robust plant growth.

Moreover, these nanoparticles can serve as carriers for pesticides or antimicrobial agents, providing targeted delivery to specific sites in plants. This could reduce the overall quantity of chemicals required, minimizing environmental impact and enhancing the sustainability of agricultural practices. Ref. [68] highlighted the potential of nanoparticles in reducing chemical usage while maintaining or improving pest and disease control efficacy.

Mg NP/GW nanoparticles could play a critical role in environmental remediation, particularly in phytoremediation. These nanoparticles can enhance the ability of plants, such as *Allium cepa*, to absorb and detoxify heavy metals and other pollutants from soil and water. This capability makes them valuable for cleaning contaminated environments. Research by [69] supports the potential of nanoparticles to assist in the detoxification processes of plants, providing an effective means of addressing pollution.

In the medical field, Mg NP/GW nanoparticles have promising applications in drug delivery systems and as antimicrobial agents. These nanoparticles can be engineered for controlled and targeted drug release, improving therapeutic outcomes while minimizing side effects. Ref. [70] discussed the advantages of using nanoparticles in drug delivery, emphasizing their ability to provide sustained and targeted release.

Additionally, due to their small size and large surface area, Mg NP/GW nanoparticles exhibit significant antimicrobial properties, making them suitable for developing new antibacterial and antifungal agents. Ref. [71] emphasized the potential of nanoparticles as a new generation of antimicrobials, particularly in combating antibiotic-resistant pathogens.

In industrial contexts, Mg NP/GW nanoparticles could be utilized as catalysts in chemical reactions, enhancing efficiency and reducing costs. Their high surface area and reactivity make them ideal candidates for catalysis in various industrial processes. Ref. [72] discussed the application of nanoparticles in catalysis, highlighting their ability to improve reaction rates and product yields.

Furthermore, these nanoparticles could contribute to the development of new composite materials with enhanced properties, such as increased strength, thermal stability, and electrical conductivity. Such materials could find applications in the electronics, construction, and aerospace industries. Ref. [73] explored the potential of nanostructured materials in advanced electrochemical energy devices, underscoring the broad applicability of nanoparticles in material science.

Nanotechnology, particularly involving Mg NP/GW nanoparticles, offers avenues for promoting sustainable agricultural and environmental practices. For example, by enhancing the efficiency of fertilizers and pesticides, these nanoparticles can help reduce the overall use of agricultural chemicals, promoting more sustainable farming practices [74]. Additionally, nanoparticles can be employed in water purification systems to remove contaminants, providing clean water for irrigation and reducing dependency on freshwater sources [75].

To fully harness the potential of Mg NP/GW nanoparticles, further research is necessary to understand their long-term effects on plants, animals, and ecosystems. Studies should focus on the degradation, accumulation, and potential toxic effects of these nanoparticles over extended periods [51]. Additionally, investigating the molecular mechanisms through which these nanoparticles interact with biological systems will provide deeper insights into their effects and help optimize their use in various applications [76].

The study of Mg NP/GW nanoparticles in *Allium cepa* root tips reveals significant cytotoxic and genotoxic effects, underscoring the need for cautious and informed application of nanotechnology. Despite these concerns, the potential applications of these nanoparticles in agriculture, environmental remediation, medicine, and industry are vast and promising. Responsible practices and comprehensive research are essential to maximize their benefits while minimizing potential risks, ensuring that nanotechnology contributes to sustainable development and technological advancement.

## 3. Materials and Methods

### 3.1. Chemicals and Reagents

The reagents and substances used in this investigation were all of the highest analytical grade. Magnesium chloride hexahydrate (MgCl_2_·6H_2_O) was sourced from Oxford Laboratory Reagent and Alpha Chemika in India. Hydrochloric acid (HCl) was supplied by Carlo Erba Reagents in Egypt. Piochem for Laboratory Chemicals in Egypt provided sodium hydroxide, iron sulfate (FeSO_4_), and sulfuric acid (H_2_SO_4_). Potassium dichromate (K_2_Cr_2_O_7_) and diphenylamine (C_12_H_11_N) were supplied by Adwic in Egypt. Glacial acetic acid (C_2_H_2_O_2_), phosphoric acid (H_3_PO_4_), and absolute ethanol (C_2_H_5_OH) were obtained from Power Chemical, Quality Control Lab in Egypt. Double-distilled water was used to prepare all solutions.

### 3.2. Collection of the Plant Material

GW was sourced from a medicinal and aromatic plants factory situated in the village of Sedmant Al-Jabal, Ehnasia Elmadina city, Beni-Suef, Egypt. *S. alba* seeds were obtained from a farm within the Faculty of Agriculture at Beni-Suef University to investigate the impact of nanoparticles on seed germination, gene expression, and growth. *P. graveolens* seedlings were acquired from a specialized farm in the new Beni-Suef city to explore the effects of nanoparticles on growth and gene expression. The influence of nanoparticles on mitotic cell division and mitotic cytotoxicity was assessed using the root tips of *Allium cepa* plants.

The collected GW was transported to the Nanotechnology Laboratory in the Faculty of Postgraduate Studies for Advanced Sciences at Beni-Suef University, Egypt, and processed promptly. GW samples without any signs of disease were carefully selected and then washed twice using bi-distilled water (DW). Subsequently, the GW was finely chopped, dried, and ground into a powder using an electric mixer. The material was then filtered through a 0.1 mm diameter screen [77]. Next, the GW powder was transformed into nano powder through ball milling (conducted by Photon Company, Egypt) for a duration of 3 h at 300 rpm. Ceramic balls with diameters ranging from 1.11 to 1.75 cm were utilized in a stainless-steel vessel measuring 7.5 cm in diameter. The waste-to-ball weight ratios were adjusted to 1:10.

### 3.3. Preparation of Mg NP/GW Nanocomposite and Zero-Valent Mg NPs

**Scheme 1:**
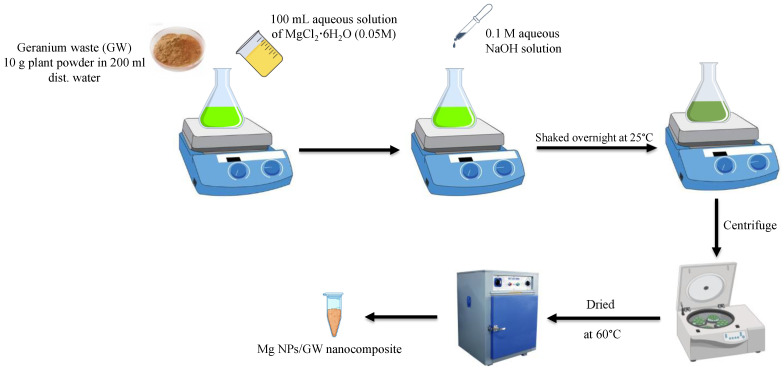
Schematic representation of Mg NP/GW nanocomposite synthesis.

To prepare the Mg NP/GW nanocomposite, 10 g of GW was suspended in 200 mL of bi-distilled water. GW served as a support to prepare this two-phase nanocomposite. To prepare the second phase, (Mg NPs), magnesium chloride was used as a precursor for a typical precipitation process. Additionally, a separate 100 mL aqueous solution of MgCl_2_·6H_2_O (0.05M) was prepared and added to the previous suspension in a 500 mL conical flask [78,79,80]. Gradually, 0.1 M aqueous NaOH solution was added drop by drop to adjust the pH to 6. This suspension was then stirred using a rotary shaker at 150 rpm at 25 °C overnight. Subsequently, centrifugation was employed to separate the produced nanocomposites, which were then subjected to repeated washing with distilled water and dried at 60 °C in an electric oven. The dried samples were finally stored for further analysis and application [81]. The process is shown in Scheme 1.

**Scheme 2:**
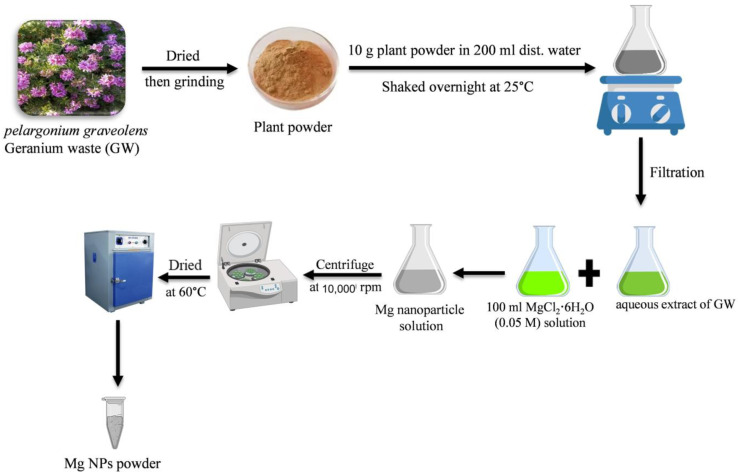
Schematic representation showing zero-valent Mg nanoparticle preparation.

Zero-valent Mg nanoparticles were prepared using a protocol similar to the one described [82]. Initially, an aqueous extract of GW was prepared by adding 10 g of GW to 200 mL of distilled water. This extract served as a precipitating and capping agent in the precipitation of the intended one-phase zero-valent Mg nanoparticles, prepared by a typical green synthesis process. The suspension was then shaken overnight using a rotary shaker at 150 rpm at 25 °C. Afterward, the suspension was filtered, and the filtrate was separated. Subsequently, a 100 mL aqueous solution of MgCl_2_·6H_2_O (0.05 M) was added to the filtrate. Dropwise addition of 0.1 M aqueous NaOH solution was carried out until the pH reached 6. Once the zero-valent magnesium nanoparticles were prepared, they were dried in a hot air oven at 60 °C and separated using centrifugation at 10,000 rpm [83]. The process is shown in Scheme 2.

### 3.4. Characterization of Nanoparticles

XRD (PANalytical Empyrean, Almelo, The Netherlands) was employed for the characterization of the produced materials. The scan angle ranged from 5 to 80° with a scan step of 0.05°, and a 30 mA current was applied with an accelerating voltage of 40 KV. The vibration of chemical bonds was analyzed using a Bruker apparatus (Vertex 70 FTIR-FT, Karlsruhe, Germany). Surface morphology of the produced materials was examined using a scanning electron microscope (SEM). Additionally, an automatic surface analyzer (TriStar II 3020, Micromeritics, Norcross, GA, USA) was utilized for determining the BET-specific surface area, pore volume, and pore size distribution of the produced materials through the N2 adsorption–desorption method. Particle sizes and zeta potentials were measured using a Malvern instrument (Malvern Instruments Ltd., Worcestershire, UK). Samples were prepared in accordance with standard protocols [84]. Thermogravimetric–differential thermal studies (TGA–DT) were conducted by TA Instruments (SDT 2960, New Castle, DE, USA). The behaviors of the produced samples were assessed in an air environment ranging from 25 °C to 1400 °C at a heating rate of 10 °C min^−1^.

The prepared samples intended for DLS analysis should be dispersed in a liquid phase. Deionized or Milli-Q water was used as the solvent in this case. Filtration may also be necessary for samples obtained in suspension to remove undesirable species with large molecular masses. It is important to ensure the suspension is homogeneous and the samples are evenly distributed. This can be achieved by gentle pipetting or, for delicate samples, by bath sonication at maximum power for up to 15 min [85].

### 3.5. Soil Analysis and Pot Experiment

During the 2023 cropping season, an experiment was conducted in the field at a private farm in New Beni-Suef City to evaluate the potential total or partial substitution of GW and Mg NP/GW by using nano-natural plants and its effect on the quality and quantity of geranium and mustard. Soil samples were collected from a clean area near Yousif Sea in Ehnasia-Elmadina city at a depth of 5 to 30 cm. These soil samples were then sieved through a 2 mm mesh to remove large debris and pebbles. Subsequently, the obtained soil samples were air-dried for a week at a temperature ranging between 21 and 24 °C [86]. Before incorporating GW and Mg NP/GW powder nanocomposite into the sieved soil, the soil was thoroughly mixed by hand. The collected soil was then transferred into plastic pots, with one kilogram of mixed soil added to each pot along with 0.5, 1.0, and 5.0 g of Mg NP/GW and GW. Growth tests were conducted in three replicates, with two seedlings of *P. graveolens* planted in each pot and five seeds of *S. alba* germinated in each pot.

To elaborate, separate handheld kitchen mixers were utilized to mechanically mix the powder nanoparticles for five minutes after their initial application to the soil at doses of 0.5, 1.0, and 5.0 g kg ^−1^ for Mg NP/GW and GW NPs. Soil without nanoparticles was employed as the control [87].

Soil pH was measured in a 1:2.5 aqueous soil extract using a pH meter (model AD8000), while electrical conductivity was determined in a 1:5 aqueous soil extract using a conductivity meter (model AD8000). Organic carbon content, determined following the method described by [88], involving the oxidation of organic carbon with potassium dichromate, was employed to determine the amount of organic carbon (OC) present. The electrical conductivity (EC) of the soil was determined using a conductivity meter, with a soil/water extract prepared at a ratio of 1:2.5 (*w*/*v*). The pH readings of the soil were found to be 7.71, 7.65, and 7.74. The corresponding EC values were 202, 197.5, and 204 µS, while the total dissolved solids (TDSs) were measured at 86.8, 84.7, and 87.6 ppm. The organic carbon (OC) content was determined to be 1.5%, 1.92%, and 1.98%. Each analytical determination was conducted in triplicate.

### 3.6. Effect of Nanoparticles on S. alba Seed Germination

The *S. alba* final germination percentage was calculated based on the total number of seeds that germinated at the conclusion of the experiment, following the International Rules for Seed Testing [89]. According to these rules [90], the following formulas were employed to compute the germination parameters [91]:

Percentage of Germination (GP%) = (Gf/n) × 100

where:

—n is the total number of seeds utilized in the test

—Gf is the total number of seeds that germinated after the experiment

Before being placed on Petri dishes, the seeds of *S. alba* underwent a sterilization process. Initially, they were sterilized for two minutes using 70% ethanol, followed by an additional three-minute sterilization using a commercial sodium hypochlorite solution (2% *v*/*v*). Subsequently, the seeds were rinsed with sterile distilled water and allowed to dry [92]. Petri dishes containing 15 mL of Mg NP/GW and GW nanomaterials were prepared, with ten seeds added to each dish. Two parameters were utilized, involving three concentrations of GW and Mg NP/GW (0.5, 1.0, and 5.0 g/L) [93]. Three replicates of the germination test were conducted for each concentration.

A single piece of filter paper was placed into each 100 mm by 15 mm Petri plate. Ten seeds were then arranged on the filter paper in each dish, ensuring a space of at least one centimeter between each seed [94]. The Petri plates were subsequently placed inside an incubator at room temperature, covered, and sealed with tape. An identical experiment was conducted as a control, excluding the use of nanoparticles.

### 3.7. Effect of Nanoparticles on P. graveolens Morphological Parameters

Eight morphological growth parameters of *P. graveolens* were recorded after 90 days of germination. These parameters were: fresh and dry shoot weight, fresh and dry root weight, shoot and root lengths, and number of leaves and branches.

### 3.8. Effect of Nanoparticles on Mitosis of Allium cepa Root Tips Using Mg NP/GW

**Scheme 3:**
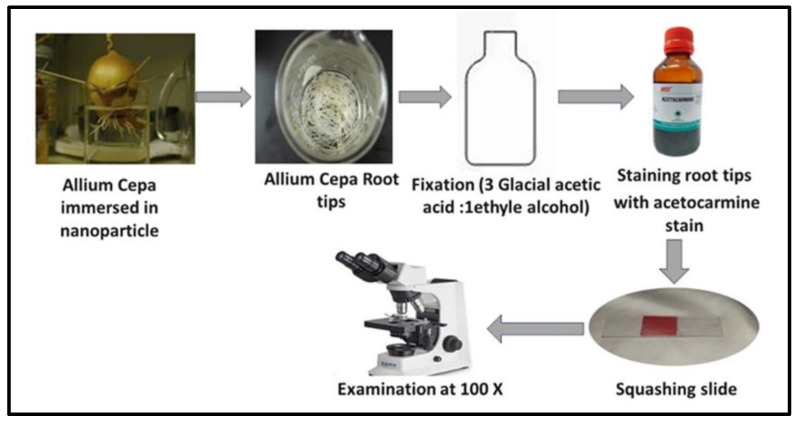
Schematic diagram for mitotic division preparation in *Allium cepa* root meristems.

Four bulbs were used for each concentration of Mg NP/GW. Before use, *Allium cepa* root tips (1–2 cm long) were preserved in 70% alcohol after treatment with Mg NP/GW. After preservation, the root tips were cleaned and fixed in a solution comprising 1 part glacial acetic acid to 3 parts ethanol for 24 h. Next, the root tips were hydrolyzed in 1.0 N HCl and then crushed in a drop of 2% acetocarmine, as illustrated in Scheme 3. A control experiment was performed without using nanoparticles [95]. To evaluate mitotic abnormalities, a total of 1000 cells were examined [96].

### 3.9. Effect of Nanoparticles on Gene Expression

The primer sequences utilized in qRT-PCR are listed in Table 7. R-T PCR was conducted with triplicate analyses using a Rotor-Gene 6000 system from Germany. Primers for the GPPS, FPPS1 precursor, and two housekeeping (or reference) genes were employed for gene expression analysis. The analysis was conducted using a SYBR^®^ Green-based methodology (Waltham, MA, USA).

The reaction mixture consisted of 2 µL of template DNA, 10 µL of SYBR Green Master Mix, 2 µL of reverse primer, 2 µL of forward primer, and sterile distilled water, resulting in a total volume of 20 µL for each reaction. The PCR assays were carried out under the following parameters: initial denaturation at 95 °C for 15 min, followed by 40 cycles of denaturation at 95 °C for 30 s and annealing/extension at 58 °C for 30 s.

The ΔCT values were calculated by subtracting the CT of the target gene from the CT of the β-Actin gene for each sample. The relative gene expression was determined using the 2-ΔΔCT method [97].

### 3.10. Statistical Analysis

For each measurement and treatment, as well as their associated controls, the means and standard deviations of three replicates were determined. Statistical analysis of the data from the different treatments was conducted using one-way analysis of variance (ANOVA), followed by mean comparison using Duncan’s test. This analysis was performed using SPSS software version 23.

## 4. Conclusions

This study investigated the Mg NP/GW nanocomposite and its effects across various parameters. FTIR spectra revealed distinctive peaks indicating functional groups such as O-H stretching vibrations, aromatic C=C stretching, amide II, and C-O bonds. SEM images confirmed aggregation of Mg nanoparticles on GW powder, and EDX analysis verified elemental composition with magnesium present at low atomic percentage (~1%). The nanocomposite exhibited a slight increase in hydrodynamic size post-Mg precipitation, suggesting aggregation or surface modifications. XRD patterns indicated a crystalline nature without significant MgO formation, suggesting surface oxide formation.

In seed germination studies, lower concentrations (0.5 g/L) of Mg NP/GW and GW enhanced seed germination rates, while higher concentrations (5.0 g/L) inhibited germination, demonstrating a concentration-dependent response. High concentrations of Mg NP/GW (5.0 g/L) showed toxicity effects on seed germination, potentially due to physiological alterations or structural interference.

In *P. graveolens*, GW (0.5 g/L) and Mg NP/GW (0.5 g/L) treatments enhanced shoot length, leaf number, and biomass, with GW showing particular effectiveness in promoting shoot length. Both treatments upregulated FPPS1 and GPPS1 gene expression in *P. graveolens* and *S. alba*, with GW NPs generally inducing higher fold changes compared to Mg NPs.

In cytogenetic studies using *Allium cepa*, lower concentrations of Mg NP/GW (0.5 g/L) increased the mitotic index, while higher concentrations (5.0 g/L) decreased it, indicating a dual effect on cell division. Mg NP/GW treatments also induced various aberrations in chromosome behavior, suggesting potential cytotoxic effects at higher concentrations.

Overall, the Mg NP/GW nanocomposite exhibited unique physical, chemical, and biological properties influenced by nanoparticle concentration and interaction with the substrate material. This study underscores the importance of dose-dependent application in agricultural practices to harness beneficial effects while mitigating potential adverse impacts.

## Figures and Tables

**Table 1:** BET surface area, total volume and area of pores, and average pore width for prepared samples.

Sample	BET Surface Area (m^2^/g)	Total Volume in Pores ≤ (cm^3^/g)	Total Area in Pores ≥ (m^2^/g)	BJH Desorption Average Pore Width (Å)
GW powder	2.7	0.00502	1.741	44.4
Mg NPs	42.2	0.165	69.250	5.1
Mg NP/GW powder	3.0	0.00537	1.885	44.2

**Table 2:** Influence of Mg NP/GW and GW concentrations on germination percentage of *S. alba* seeds *.

NP Concentration (g/L)	Control	Mg NP/GW			GW		
GP%		**0.5**	**1.0**	**5.0**	**0.5**	**1.0**	**5.0**
	100.00 ± 0.0	100 ± 0.0	93.33 ± 0.58	46.67 ± 1.53	96.67 ± 0.58	96.67 ± 0.58	83.30 ± 0.5

* Representing significant effects at a 0.05% probability level. GP = germination percentage.

**Table 3:** Effects of different concentrations of Mg NP/GW and GW nanoparticles on shoot length, root length, number of branches, number of leaves, and fresh and dry weight of *P. graveolens* plant *.

Treatment (g/L)	Shoot	Root	Leaves	Branches	Fresh Weigh		Dry Weigh	
	Length (cm)	Length (cm)	Number NO.	Number NO.	Shoot (g)	Root (g)	Shoot (g)	Root (g)
**Control**	18.00 ± 1.26 (a)	16.67 ± 1.65 (a)	28.08 ± 6.30 (a)	3.33 ± 0.33 (a)	19.63 ± 1.35 (a)	1.42 ± 0.19 (a)	5.27 ± 0.43 (a)	0.53 ± 0.10 (ab)
**Mg** **NP/GW 0.5**	23.67 ± 0.80 (c)	26.33 ± 0.49 (b)	52.50 ± 5.76 (c)	4.17 ± 0.31 (ab)	19.12 ± 4.35 (a)	1.28 ± 0.18 (a)	4.86 ± 1.15 (a)	0.45 ± 0.06 (a)
**Mg** **NP/GW 1**	20.67 ± 0.42 (abc)	24.50 ± 1.28 (b)	44.83 ± 2.34 (bc)	3.00 ± 0.26 (a)	21.79 ± 2.96 (a)	2.09 ± 0.35 (a)	5.93 ± 0.39 (a)	0.77 ± 0.07 (ab)
**Mg** **NP/GW 5**	19.50 ± 0.72 (ab)	20.33 ± 1.82 (a)	31.50 ± 2.94 (ab)	3.00 ± 0.45 (a)	25.21 ± 0.77 (a)	1.99 ± 0.21 (a)	6.07 ± 0.56 (a)	0.70 ± 0.08 (b)
**GW** **0.5**	30.83 ± 1.30 (d)	25.00 ± 1.06 (b)	53.83 ± 4.27 (c)	4.67 ± 0.33 (b)	23.34 ± 1.66 (a)	1.55 ± 0.05 (a)	6.49 ± 0.31 (a)	0.71 ± 0.05 (ab)
**GW** **1**	22.67 ± 2.08 (bc)	20.33 ± 1.15 (a)	36.17 ± 5.59 (ab)	3.33 ± 0.33 (a)	22.61 ± 0.47 (a)	1.87 ± 0.42 (a)	5.47 ± 0.34 (a)	0.65 ± 0.12 (ab)
**GW** **5**	23.00 ± 0.63 (bc)	25.67 ± 1.33 (b)	34.83 ± 4.63 (ab)	3.83 ± 0.48 (ab)	22.93 ± 0.37 (a)	1.30 ± 0.42 (a)	5.13 ± 0.54 (a)	0.63 ± 0.10 (ab)

* Different letters (a–d) denote significant differences between treatments at *p* < 0.05.

**Table 4:** Effects of Mg NP/GW on mitotic abnormalities and cell division in *Allium cepa*.

Replicates	Concentration of Mg NP/GW (g/L)	Number of Counted Cells	Normal Metaphase	Normal Anaphase	Sticky Chromosome	Laggard Chromosome	Chromosomal Bridge	Disturbed Metaphase
Control	0.0	1000	+	+	−	−	−	−
Nanoparticle (0.5 g/L)	0.5	1000	+	+	+	+	−	−
Nanoparticle (1.0 g/L)	1.0	1000	+	+	+	−	+	+
Nanoparticle (5.0 g/L)	5.0	1000	+	−	+	+	+	+
**Condition**	**Concentration of Mg NP/GW (g/L)**	Mean ± SE (%) *	Mitotic Index (%) **	**Telophase**	**Anaphase**	**Metaphase**	**Prophase**	**Dividing Cell (Total)**
Control	0.0	44.6 ± 2.16	45.8–50.1	0–2	1–5	2–4	380–493	386–501
Nanoparticle (0.5 g/L)	0.5	61.24 ± 1.79	54.6–64.5	0–3	1–8	4–16	530–640	546–645
Nanoparticle (1.0 g/L)	1.0	36.96 ± 0.86	34.6–39.2	0–1	1–3	1–2	340–390	346–392
Nanoparticle (5.0 g/L)	5.0	27.36 ± 1.34	24.1–31.7	0	0–1	0–2	240–315	241–317

(+) = present. (−) = absent. * Mean ± SE (%) = average (mitotic index (%)). ** Mitotic index (%) = dividing cell (total)/1000 × 100.

**Table 5:** Relative gene expression level (fold changes) of FPPS1 gene in plants growing under nanoparticles application; data values expressed as (Mean ± SD).

Treatment	Plant	Relative Expression (Fold Changes)
Control	*S. alba*	1.0 ± 0.03 ^d^
	*P. graveolens*	1.0 ± 0.03 ^d^
GW NPs (0.5 g/L)	*S. alba*	25.244 ± 0.004 ^a^
	*P. graveolens*	21.58 ± 0.03 ^b^
Mg NPs (0.5 g/L)	*S. alba*	13.4539 ± 0.002 ^c^
	*P. graveolens*	12.15676 ± 0.0025 ^c^

* Different letters (a–d) denote significant differences between treatments at *p* < 0.05.

**Table 6:** Relative gene expression level (fold changes) of GPPS1 gene in plants growing under nanoparticles application; data values expressed as (Mean ± SD).

Treatment	Plant	Relative Expression (Fold Changes)
Control	*S. alba*	1.0 ± 0.03 ^e^
	*P. graveolens*	1.0 ± 0.03 ^e^
GW NPs (0.5 g/L)	*S. alba*	18.1340 ± 0.002 ^a^
	*P. graveolens*	14.574 ± 0.003 ^b^
Mg NPs (0.5 g/L)	*S. alba*	11.273 ± 0.04 ^c^
	*P. graveolens*	6.477 ± 0.004 ^d^

* Different letters (a–e) denote significant differences between treatments at *p* < 0.05.

**Table 7:** Oligonucleotide primers used in qRT-PCR analysis.

Gene Name		Primer Sequence	Accession No.	Tm (°C)
**FPPS1**	F	5′-CCGATGATTCTCGCCAATGGG-3′	At5g47770	58 °C
	R	5′-CCATTCAATGCACCAACCAAGTG-3′		
**GPPS1**	F	5′-CCAAATGCCTCGTCATCT-3′	At5g49530	
	R	5′-ATTAGAGTCAAGCTCAAAAGG-3′		
**β-Actin**	F	5′-GTGCCCATTTACGAAGGATA-3′	AB181991	
	R	5′-GAAGACTCCATGCCGATCAT-3′		
**GAPDH**	F	5′-TTGGTTTCCACTGACTTCGTT-3′	CA254672	
	R	5′-CTGTAGCCCCACTCGTTGT-3′		

## Data Availability

The authors confirm that the data supporting the findings of this study are available within the article.

## Supplementary Materials

The following supporting information can be downloaded at: https://www.mdpi.com/article/10.3390/molecules29143394/s1, Table S1: Effects of Mg NPs/GW on mitotic abnormalities in *Allium cepa*; Table S2: Effects of Mg NPs/GW on mitotic cell division in *Allium cepa*.
