# Supplementary material for: Unveiling the Impact of Eco-Friendly Synthesized Nanoparticles on Vegetative Growth and Gene Expression in Pelargonium graveolens and Sinapis alba L
Source: Molecules. 2024 Jul 19;29(14):3394. doi: 10.3390/molecules29143394 (PMC11280068; doi:10.3390/molecules29143394)
Supplement: Supplementary file 1 [file molecules-29-03394-s001.zip › molecules-3072541-supplementary.pdf]

Table S1. Effects of Mg NPs/GW on mitotic abnormalities in *Allium cepa.*

Replicates	Concentration of Mg NPs/GW (g/L)	Number of counted cells	Normal metaphase	Normal anaphase	Sticky chromosome	Laggard Chromosome	Chromosomal bridge	Disturbed metaphase
**R 1**	Control	1000	+	+	_	_	_	_
**R 2**	(deionized	1000	+	+	_	_	_	_
**R 3**	distilled	1000	+	+	_	_	_	_
**R 4**	water)	1000	+	+	_	_	_	_
**R 5**		1000	+	+	_	_	_	_
**R 1**		1000	_	+	_	+	+	+
**R 2**	Nanoparticle	1000	+	+	+	_	_	+
**R 3**	(0.5)	1000	_	+	+	+	_	_
**R 4**		1000	+	+	_	_	+	_
**R 5**		1000	+	+	+	_	_	+
**R 1**	Nanoparticle	1000	+	+	+	_	+	+
**R 2**	(1.0)	1000	+	+	+	+	+	+
**R 3**		1000	+	+	+	+	_	+
**R 4**		1000	+	+	+	+	_	_
**R 5**		1000	+	+	+	_	+	+
**R 1**	Nanoparticle	1000	+	+	+	+	+	+
**R 2**	(5.0)	1000	+	_	+	+	+	+
**R 3**		1000	+	_	+	+	+	+
**R 4**		1000	_	_	+	+	+	+
**R 5**		1000	+	_	+	+	+	+

Table S2. Effects of Mg NPs/GW on mitotic cell division in Allium cepa

Replicates	Concentration of Mg NPs/GW (g/L)	Dividing cell (total)	Prophase	metaphase	Anaphase	Telophase	Mitotic index (%) *	Mean± SE (%) **
**R 1**	Control	458	450	3	5	0	45.8	44.6±
**R 2**	(deionized	480	471	4	4	1	48.0	2.16%
**R 3**	distilled	386	380	2	3	1	38.6	
**R 4**	water)	501	493	3	5	0	50.1	
**R 5**		405	400	2	1	2	40.5	
**R 1**		546	530	12	4	0	54.6	61.24±
**R 2**	Nanoparticle	627	600	16	8	3	62.7	1.79%
**R 3**	(0.5)	605	590	11	3	1	60.5	
**R 4**		639	625	9	4	1	63.9	
**R 5**		645	640	4	1	0	64.5	

**R 1**	Nanoparticle	373	370	1	2	0	37.3	36.96±
**R 2**	(1.0)	392	390	1	1	0	39.2	0.86%
**R 3**		354	350	2	1	1	35.4	
**R 4**		346	340	2	3	1	34.6	
**R 5**		383	380	1	2	0	38.3	
**R 1**	Nanoparticle	282	280	1	1	0	28.2	27.36±
**R 2**	(5.0)	278	277	1	0	0	27.8	1.34%
**R 3**		317	315	2	0	0	31.7	
**R 4**		250	250	0	0	0	25.0	
**R 5**		241	240	1	0	0	24.1	
